# 

**Published:** 1894-07

**Authors:** 


### CONTENTS FOR JULY, 1894.

| ORIGINAL COMMUNICATIONS. | PAGE. |
|---|---|
| The Value of Hegar's Sign of Pregnancy. By J. W. LONG, M. D. | 705 |
| What Kills the Patient—Peritonitis or Infection? By BYRON ROBINSON | 711 |
| Bladder Gymnastics and Auto-Irrigation. By BYRON H. DAGGETT, M. D. | 714 |
| **CLINICAL REPORTS.** | |
| Clinical Memoranda from the Sisters' of Charity Hospital | 718 |
| Puerperal Convulsions with Albuminuria | 723 |
| **SOCIETY PROCEEDINGS.** | |
| The Medical Society of the County of Erie | 725 |
| **SELECTIONS.** | |
| Surgical Problems in Intra-Pelvic and Abdominal Diseases | 727 |
| A Case of Extirpation of the Rectum by the Kraske Method | 732 |
| Hematoma of the Ovary | 733 |
| Breech Presentations | 738 |
| **EDITORIAL.** | |
| The National Medical Societies | 739 |
| Topics of the Month | 741 |
| Obituary | 744 |
| Personal | 747, 764 |
| Society Meetings | 749, 764 |
| Hospital Notes | 750 |
| Academy of Medicine Notes | 750 |
| Medical College Notes | 751 |
| Book Reviews | 752 |
| Literary Notes | 765 |

